# Dysbiosis in a canine model of human fistulizing Crohn’s disease

**DOI:** 10.1080/19490976.2020.1785246

**Published:** 2020-07-30

**Authors:** Ana Maldonado-Contreras, Lluís Ferrer, Caitlin Cawley, Sarah Crain, Shakti Bhattarai, Juan Toscano, Doyle V. Ward, Andrew Hoffman

**Affiliations:** a Department of Microbiology and Physiological Systems, University of Massachusetts Medical School, Worcester, MA, USA; b Cummings School of Veterinary Medicine, Tufts University, North Grafton, MA, USA

**Keywords:** fistulizing Crohn’s disease, microbiome, dysbiosis, perianal fistulas, canine furunculosis

## Abstract

Crohn’s disease (CD) is a chronic immune-mediated inflammatory condition caused by the loss of mucosal tolerance toward the commensal microbiota. On average, 29.5% and 42.7% CD patients experience perianal complications at 10 and 20 y after diagnosis, respectively. Perianal CD (pCD) result in high disease burden, diminished quality of life, and elevated health-care costs. Overall pCD are predictors of poor long-term outcomes. Animal models of gut inflammation have failed to fully recapitulate the human manifestations of fistulizing CD. Here, we evaluated dogs with spontaneous canine anal furunculosis (CAF), a disease with clinical similarities to pCD, as a surrogate model for understanding the microbial contribution of human pCD pathophysiology.

By comparing the gut microbiomes between dogs suffering from CAF (CAF dogs) and healthy dogs, we show CAF-dog microbiomes are either very dissimilar (dysbiotic) or similar (healthy-like), yet unique, to healthy dog’s microbiomes. Compared to healthy or healthy-like CAF microbiomes, dysbiotic CAF microbiomes showed an increased abundance of *Bacteroides vulgatus* and *Escherichia coli* and a decreased abundance of *Megamonas* species and *Prevotella copri*.

Our results mirror what have been reported in previous microbiome studies of patients with CD; particularly, CAF dogs exhibited two distinct microbiome composition: dysbiotic and healthy-like, with determinant bacterial taxa such as *E. coli* and *P. copri* that overlap what it has been found on their human counterpart. Thus, our results support the use of CAF dogs as a surrogate model to advance our understanding of microbial dynamics in pCD.

## Introduction

Inflammatory bowel diseases (IBD), including Crohn’s disease (CD), are immune-mediated inflammatory condition affecting more than a million individuals in the US. On average, 29.5% and 42.7% CD patients experience perianal complications at 10 and 20 y after diagnosis, respectively.^1,2^ Perianal Crohn’s disease, or pCD, represents one of the most clinically significant complications and is a predictor of poor long-term outcome in CD.^3^ Perianal fistulas are most common in patients suffering from colonic CD with rectal involvement (L2). The etiology of pCD is not well defined yet is thought to be a combination of genetic and immune factors, and the microbiome. Several genetic variants have been also associated with increased risk of development of pCD, specifically PRDM1, NOD2, and ATG16L1.^4,5^ Immunologically, patients with pCD exhibit an up-regulated expression of TNF-α compared to CD patients without perianal complications or healthy controls.^6^ The role of the microbiome has gained attention in recent years as evidence indicating its involvement emerges.^7–9^ Particularly, treatment with antibiotics (reviewed in^2,10^), fecal diversion,^7,8^ and fecal transplants^9^ have proven to be effective for managing the disease. Definition of the pathogenesis and pathophysiology of pCD is key to further advance treatment strategies for patients. One of the main challenges of pCD research is the lack of a relevant, reliable, and reproducible animal model. Here, we aim to evaluate the usefulness of dogs as a model system to elucidate the contribution of the microbiota to the pathophysiology of pCD.

We have postulated that canine anal furunculosis (CAF) can be used as a model for studying the pathophysiology and treatment of pCD.^11,12^ CAF and pCD exhibit common clinical signs such as focal or multifocal dissecting sinus tracts (external openings) and ulcerations on the perianal area. The American Gastroenterological Association proposed a classification of “simple” and “complex” fistulas^13^ in pCD. Simple fistulas include superficial, intersphincteric, or intrasphincteric fistulas below the dentate line, with a single tract, and absence of perianal complications. Complex fistulas stretch above the dentate line (intersphincteric, transphincteric, extrasphincteric, suprasphincteric), with many tracts, and may be related to perianal abscesses, rectal stricture, proctitis, or can extend to the bladder or vagina.^1,14^ Similarly, CAF includes epithelial-lined single or multiple (such as complex fistulas in pCD) tracts that develop in the perianal tissue including superficial, intersphincteric, or intrasphincteric fistulas that can extend to the rectal lumen^15,16^ but not to other areas (compared to simple fistulas in pCD).

Treatment for both CAF and pCD requires a combination of immunosuppressive drugs (i.e., cyclosporin, and tacrolimus),^17^ adjuvant antibiotic therapy, and/or surgery (i.e., fistulectomy, fistulotomy, drainage). Cyclosporin has been effective in managing both pCD and CAF with up to 78% and 100% of human (reviewed^18^) and dogs (reviewed^19^) responding to treatment, respectively. In addition, positive clinical response to tacrolimus is similar in pCD^18^ and dogs with CAF^19^ (44–78% and 50%, respectively). Antimicrobials alone do not seem effective for the management of pCD nor for CAF. In humans, antibiotics are administrated as initial treatment or in combination with other therapies such as immunosuppressants.^2^ A few studies have reported the use of metronidazole in combination with other therapies (azathioprine, tacrolimus ointment, prednisone) for the treatment of CAF.^20,21^

Treatment with diet for both pCD and CAF is still in debate. A dietary guidance for patients with IBD has been recently published highlighting the lack of strong evidence for foods to include or avoid for patients with IBD.^22^ Most IBD-friendly whole food diets involved exclusion of carbohydrates, inclusion of lean protein, fruits, and vegetables.^23–25^ Similarly, few studies have demonstrated adverse reaction of foods in some dogs with CAF.^21,26-28^ Dietary treatment with “novel protein” -refer to foods with protein sources not consumed before by the dog with CAF- such as venison, lamb, fish, bison, duck-show modest results in CAF treatment when used in combination with immunosuppressants, antibiotics, and/or surgery.^21,26-28^

Despite of having positive clinical responses to the aforementioned treatments, the majority of both pCD and CAF patients experience relapses of the perinal manifestations.

Of note, the German Shepherd Dog (GSD) appears to be overrepresented within the population of dogs with CAF, with more than 80% of dogs suffering from CAF (CAF dogs) being GSD.^29,30^ GSD is also susceptible to inflammatory bowel disease (IBD),^31,32^ systemic aspergillosis,^33–36^ and deep pyoderma,^37,38^ suggesting that GSD might have a broadly dysfunctional immune response to microbial exposure at epithelial surfaces, similar to human CD patients. Dogs have already proven useful for studying various other spontaneously occurring disorders similar to those affecting humans,^11,39-42^ and the dog microbiome is more similar to that of humans than it is to the microbiomes of other animals that are generally used as models of microbiome-centered diseases such as IBD (e.g., mice and pigs).^43^ Dogs also spontaneously develop IBD^31^ and can experience common disease complications, including perianal fistulas,^16^ and exhibit IBD-associated microbiome alterations that resemble changes observed in humans with IBD.^44,45^ Thus, dogs represent an ideal model for studying IBD and in particular pCD.

Given that the intersection of immunity and the microbiota seems to be at the heart of both pCD and CAF, we sought to explore naturally occurring CAF as a surrogate model for human pCD. The aim of this study was to characterize the bacterial microbiome structure of dogs suffering from CAF. We posit that characterizing the microbiomes of CAF dogs will help clarify the pathophysiology of CAF and advise on the translational significance of CAF as an animal model for pCD.

## Results and discussion

### CAF is associated with gut microbiota changes

**Figure 1.**
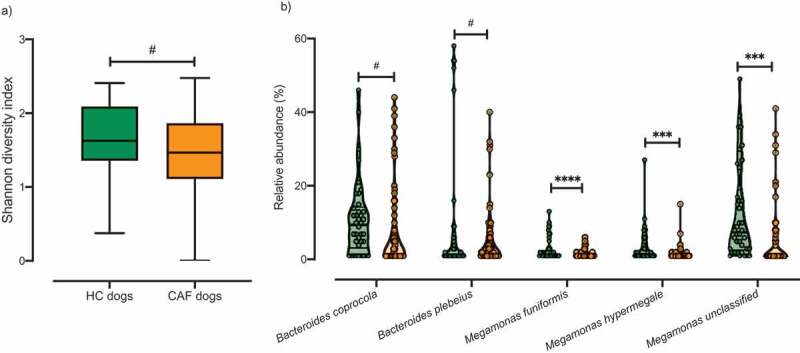
Analysis of the microbiota composition in samples from CAF dogs and HC dogs. A) Shannon index of alpha diversity for HC dogs (green) and CAF dogs (orange). B) Relative abundance of the bacterial taxa found to discriminate between dogs with different disease status: HC dogs (green) and CAF dogs (orange); thick black lines indicate mean abundance. *T*-test, *p* values are expressed as: ****<0.0001, ***<0.001, and #0.1 (Corrected for multiple comparisons (5 bacterial taxa found significantly different by disease status) using the Bonferroni-Dunn method, with alpha = 0.05).

A total of 20 dogs were recruited for this study: eight healthy control dogs (HC dogs), and 12 CAF dogs (**Table 1**). We collected 4–6 fecal samples per dog, totaling 70 CAF samples and 38 HC samples. Shotgun sequencing of the samples revealed the presence of the following bacterial phyla (relative abundance indicated as percentage of total reads): *Firmicutes* (48%), *Bacteroidetes* (31%), *Proteobacteria* (12%), *Actinobacteria* (7%), and *Fusobacteria* (2%). When we analyzed differences in microbial composition between dogs according to their disease status, we found slightly lower alpha diversity in CAF dogs compared to HC dogs (Figure 1A, Shannon diversity, Kruskal–Wallis test, *p* value = .06), similar to previous results with IBD dogs.^46^ As expected, there was also generally lower alpha diversity in older dogs compared to younger dogs (Spearman correlation, *p* value = .1). No significant differences in alpha diversity were observed by weight.

**Table 1.** Characteristics of the recruited dogs according to disease status and cluster.

	HC dogs (n = 8, 38 samples)	CAF dogs (n = 12, 70 samples)	CAF dogs, near cluster (n = 7, 65 samples)	CAF dogs, far cluster (n = 5, 43 samples)
**Dogs description**				
Average age (years) ± STDEV	7.3 ± 1.43	4.3 ± 1.28	7.9 ± 1.53	7 ± 0.89
Average weight (kg) ± STDEV	36.9 ± 7.93	41.1 ± 7.31	42.4 ± 9.37	39.2 ± 2.77
Female sex	4 (50%)	3 (25%)	1 (14.3%)	2 (40%)
**Number of fistulas**				
0–2	0	4	3	1
3–5	0	5	3	2
>5	0	3	0	3
**Pathology findings**				
*Morphological features*	ND			
Crypt hyperplasia		3 (25%)	1 (14.3%)	2 (40%)
Crypt dilation/distortion (% positive)		8 (66.7%)	5 (71.4%)	3 (60%)
Fibrosis/atrophy (% positive)		8 (66.7%)	5 (71.4%)	3 (60%)
*Inflammation*	ND			
Lamina Propria lymphocytes and plasma cells (% positive)		3 (25%)	1 (14.3%)	2 (40%)
Lamina Propria Eosinophils (% positive)		4 (33.3%)	2 (28.6%)	2 (40%)
*Final diagnosis*	ND			
Normal mucosal (% positive)		3 (25%)	2 (28.6%)	1 (20%)
Lymphoplasmatic inflammatory (% positive)		2 (16.7%)	0	2 (40%)
Eosinophilic inflammatory (% positive)		4 (33.3%)	2 (28.6%)	2 (40%)
Mucosal atrophy/fibrosis (non-inflammatory) (% positive)		5 (41.7%)	3 (42.9%)	2 (40%)

After filtering out low abundance genera (i.e., genera not detected four times or more in at least 20% of the samples), we performed gneiss analysis,^47^ which applies the concept of balance trees to compositional data to identify microbial subcommunities that covary with environmental variables. The gneiss analysis considers the log-ratio abundances of subcommunities within the microbiome to indicate taxa whose abundances change relative to other taxa with respect to a variable of interest; in our analysis, the variable of interest was disease status (CAF versus HC). We used linear mixed-effect models to account for inter-individual microbiome variability in this analysis. When we analyzed the microbiomes according to disease status, we found that *Megamonas hypermegale, Megamonas unclassified, Bacteroides coprocola, Megamonas funiformis*, and *Bacteroides plebeius* were differentially abundant in CAF dogs compared to HC dogs (**Table 2**). We further confirmed that there were significant differences in the proportions of these five bacterial taxa between HC dogs and CAF dogs, with lower abundance in CAF dogs (Figure 1B, false discovery rate [FDR], *t*-test). We also applied analysis of the composition of microbiomes (ANCOM) to identify discriminant bacterial taxa for CAF dogs versus HC dogs. We identified *M. funiformis* (W = 51), *M. hypermegale* (W = 52), and *M. unclassified* (W = 60) as discriminating between CAF dogs and HC dogs. Altogether, these two robust analyses concurred that the abundance of *Megamonas* species can discriminate between HC dog samples and CAF-dog samples.

**Table 2.** Bacterial taxa with significantly different dominance between samples grouped by disease status or cluster.

Comparison	Determinant bacterial taxa	gneiss, FDR-corrected p value	Group showing increased abundance
HC dogs *versus* CAF dogs	*Bacteroides plebeius*	0.00137614	HC dogs
	*Megamonas hypermegale*		
	*Bacteroides coprocola*		
	*Megamonas unclassified*		
	*Megamonas funiformis*		
HC dogs *versus* CAF dogs in near cluster	*Megamonas funiformis*	0.01357096	HC dogs
	*Megamonas unclassified*		
CAF dogs in near cluster *versus* CAF dogs in far cluster	*Bacteroides plebeius*	0.00001034	CAF dogs in near cluster
	*Megamonas hypermegale*		
	*Bacteroides coprocola*		
	*Megamonas unclassified*		
	*Megamonas funiformis*		

### Dogs suffering from CAF exhibit a dichotomous bacterial community structure

**Figure 2.**
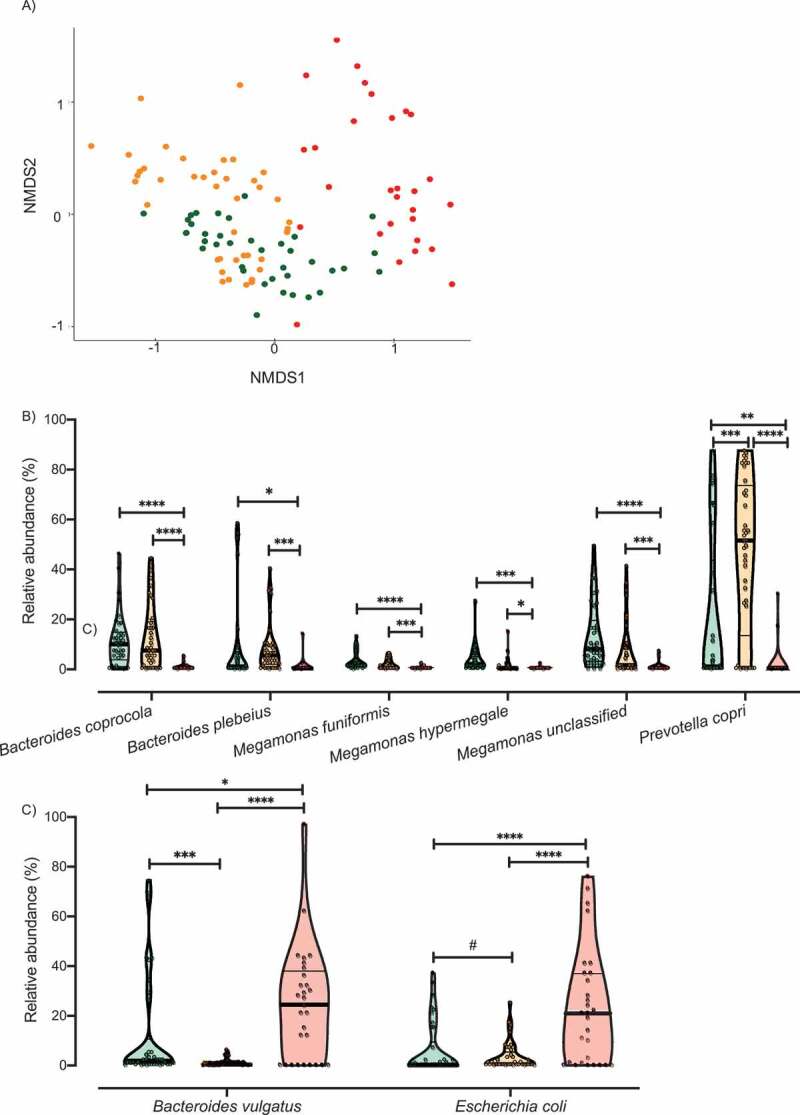
Analysis of the microbiota clusters. A) NMDS visualization of samples from HC and CAF dogs. Bacterial taxa present were quantified by MetaPhlAn, distances were calculated using Bray Curtis dissimilarity index, and samples were plotted based on NMDS. Clusters were defined using PAMK and are colored green (HC dogs), orange (CAF dogs in the near cluster) and red (CAF dogs in the far cluster). Relative abundance of the bacterial taxa found to discriminate between dogs, separated by cluster assignment: B) Bacterial taxa overrepresented in HC dogs or CAF dogs in the near cluster compared to CAF dogs in the far cluster; C) Bacterial taxa overrepresented in CAF dogs in the far cluster compared to HC dogs or CAF dogs in the near cluster. Clusters are colored as described for panel A, and thick black lines indicate mean abundance. FDR *t*-test, *p* values are expressed as: ****<0.0001, ***<0.001, ** <0.005, * <0.05, and #0.1 (Corrected for multiple comparisons (8 bacterial taxa found significantly different among clusters and HC) using the Bonferroni-Dunn method, with alpha = 0.05).

We, next, investigated the microbiome patterns further. Using partitioning around medoids with the estimation of the number of clusters (PAMK), we determined the optimal number of clusters (Supplementary Figure 1) based on the Bray Curtis dissimilarity index calculated from the relative abundance of each bacterial taxa (n = 61). Clustering results are visualized the with non-metric multidimensional scaling (NMDS). As seen in Figure 2A, CAF dogs were divided into two clusters: healthy-like cluster, referred to hereafter as “near cluster,” gathering in close proximity to HC dogs; and dysbiotic cluster, referred to hereafter as “far cluster,” positioned significantly more distant from the HC dogs (Bray Curtis distance, PERMANOVA, R^2^ = 0.252, *p* value = .001. Supplementary Figure 2). Although the average silhouette width in this analysis indicated a weak structure, this differentiation has been repeatedly reported in patients suffering from CD.^44,48-50^ Taken together, these data suggest that the microbiota of CAF dogs can range from one that closely resembles that of a healthy individual to a dysbiotic microbiota that differs markedly from that of a healthy individual, similar what it was been observed repeatedly in CD patients.

To identify determinant taxa associated with each cluster, we performed additional analyses with gneiss and ANCOM. We found that CAF dogs belonging to the near cluster had different dominance of *M. funiformes* and *Megamonas unclassified* compared to HC dogs (**Table 2**). Moreover, ANCOM results identified *P. copri* (W = 53) as a bacterial taxon that differentiated between CAF dogs belonging to the near cluster and HC dogs.

When comparing CAF dogs belonging to the far cluster and HC dogs, both gneiss and ANCOM identified *B. coprocola* (W = 52), *M. funiformis* (W = 49), *M. hypermegale* (W = 48), and *M. unclassified* (W = 52) as distinguishing between the two groups (**Table 2**). One additional bacterial taxon was identified as being differentially dominant between CAF dogs belonging to the far cluster and HC dogs only by gneiss: *B. plebeius*.

We also investigated differences between CAF dogs belonging to the near and far clusters. We found that the two clusters differed in the dominance of *M. hypermegale, M. unclassified, B. coprocola, M. funiformis*, and *B. plebeius* (**Table 2**). Moreover, ANCOM results identified six bacterial taxa that distinguished between the near and far clusters, specifically, *B. coprocola* (W = 50), *B. plebeius* (W = 51), *Bacteroides vulgatus* (W = 52), *P. copri* (W = 53), *Megamonas unclassified* (W = 48), and *Escherichia coli* (W = 48). Particularly, we found that the relative abundance of six of those bacterial taxa decreased (Figure 2B) while two increased (Figure 2C) in the far cluster compared to HC dogs and the CAF dogs in the near cluster.

Overall, these analyses established that higher abundance of *Megamonas spp., P. copri, B. coprocola*, and *B. plebeius* is associated with healthy and healthy-like (near cluster CAF-dog) samples. On the other hand, increased abundance of *E. coli* and *B. vulgatus* is associated with dysbiosis (far cluster CAF dogs).

### Clinical manifestations of CAF are associated with dysbiosis

**Figure 3.**
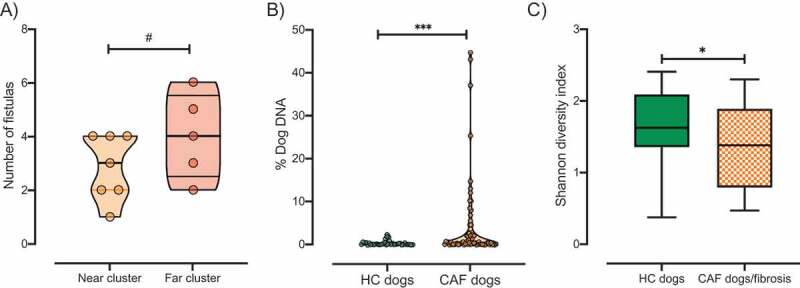
Clinical manifestation of CAF. A) The number of fistulas presented by CAF dogs according to cluster (orange, near cluster; red, far cluster). B) Percentage of dog DNA reads in metagenomic sequencing samples grouped by disease status. C) Shannon index of alpha diversity in samples from HC dogs (green, n = 8) or CAF dogs exhibiting fibrosis (orange pattern, n = 6). *T*-test, *p* values are expressed as: ****<0.0001, ***<0.001, ** <0.005, * <0.05, and #0.1.

CAF has a clinical appearance similar to that of perianal fistulas in humans, a complication frequently associated with CD. Specifically, dogs suffering from CAF develop epithelial-lined sinus tracts around the perianal tissue including superficial, intersphincteric, or intrasphincteric fistulas that can extend to the rectal lumen.^15,16^ These ulcerative tracts vary in diameter, depth, and connectivity, and can affect the entire area around the anus. These lesions are usually referred to as fistulas. The CAF dogs recruited in this study presented with varying numbers of fistulas (average, 3.3 ± 1.4. **Table 1**) but no anal sac disease was observed. We found that CAF dogs in the far cluster exhibited a trend of higher average number of fistulas than those in the near cluster (4 vs. 2.87, respectively, Figure 3A). Moreover, *Helicobacter canis* dominance was statistically different between CAF dogs according to fistula number (gneiss, FDR corrected *p value* <.0001). To determine the direction of dominance, we performed correlation analysis between *H. canis* relative abundance and number of fistulas. We found a negative correlation between *H. canis* relative abundance and the number of fistulas in CAF dogs (**Table 3**).

**Table 3.** Bacterial taxa that correlate with clinical manifestations of CAF.

Disease manifestation/assessment	Determinant bacterial taxa	Spearman correlation	*p* value	gneiss, FDR-corrected *p* value
**Gross examination**				
Number of fistulas	*Helicobacter canis*	−0.4715	< 0.001	0.00000003
**Pathology findings**				
CAF dogs with a combination of crypt hyperplasia, crypt dilation/distortion, and/or fibrosis/atrophy (n = 7)	*Ruminococcus torques*	−0.355	0.002	< 0.05
CAF dogs with a combination of microarchitectural changes and presence of inflammatory markers (n = 5)	*Subdoligranulum unclassified*	−0.1639	0.1751	0.02305158
	*Paraprevotella unclassified*	−0.3336	0.0048

It has been suggested that increased host DNA content in fecal samples may be an indicator of inflammation-associated shedding of epithelial or blood cells. Indeed, a previous study found high levels of human DNA in stool samples from pediatric patients with active CD.^44^ Thus, we determined the abundance of dog DNA sequences present in each stool sample analyzed as a percentage of total reads. We found that samples from CAF dogs contained a higher percentage (mean = 4.09%, minimum = 0.02%, maximum = 44.68%) of host DNA sequences than did samples from HC dogs (Mean = 0.41%, minimum = 0.02%, maximum = 2.38%, Mann–Whitney test, *p* value *=* .001, Figure 3B). There was no significant difference between the abundance of host DNA sequences recovered in samples from the near versus far cluster. This result suggests that, compared to their healthy counterparts, CAF dogs have a higher degree of shedding of epithelial or blood cells associated with inflammation, similar to what has been previously observed in CD humans.

In addition to fistulas, GSD with CAF frequently present clinical and histologic evidence of colitis. The major microarchitectural changes accompanying colonic inflammation in dogs include crypt hyperplasia, dilation/distortion, and mucosal atrophy or fibrosis.^51^ Thus, we also assessed morphological features and other immune-associated inflammatory markers on the colonic mucosa of the CAF dogs (**Table 1**).

First, we observed that there was a positive correlation between histopathology scores and number of fistulas (Spearman correlation, *p value* <.05). Further, we found that 75% of the CAF dogs included in this study exhibited morphological evidence of either cryptal hyperplasia, dilation/distortion, or fibrosis. CAF dogs with those major microarchitectural changes showed differential dominance of the same bacterial taxa that were associated with general CAF disease status, namely, *Megamonas* species *(M. hypermegale, M. unclassified*, and *M. funiformis), B. plebeius*, and *B. coprocola* (data not shown). In addition to these taxa, *Ruminococcus torques*, known to be decreased in CD,^44,48,52,53^ was also identified as a determinant taxon between CAF dogs with major microarchitectural changes and HC dogs (gneiss, FDR corrected *p* value <.05). Moreover, we found that *R. torques* relative abundance is negatively correlated with microarchitectural changes (**Table 3**). Interestingly, CAF dogs presenting fibrosis (50%) exhibited significantly decreased alpha diversity compared to HC dogs (Figure 3C, Shannon diversity, Kruskal–Wallis test, *p* value <.05).

Furthermore, CAF dogs exhibiting major microarchitectural combined with infiltration of immune cells (i.e., eosinophils, lymphocytes, or plasma cells) in the lamina propria (41.6%) showed differential abundance of *Subdoligranulum unclassified* and *Paraprevotella unclassified* when compared to CAF dogs without those clinical manifestations (gneiss, FDR corrected *p* value = .02). Particularly, those bacterial taxa were negatively correlated with both major microarchitectural changes and immune cell infiltration (**Table 3**). Recently, depletion of *Subdoligranulum* species in dysbiotic CD patients was associated with bile acid dysregulation.^50^

Based on the presence of major microarchitectural and immune cells in the lamina propria we calculated pathology scores as described before.^51^ We observed that the pathology scores from CAF dogs belonging to the near cluster (median = 2, minimum = 0, maximum = 5) were comparable to those of the far cluster (median = 3, minimum = 0, maximum = 5, Mann–Whitney test, *p* value >.05).

To investigate whether CAF dogs exhibited peripheral signs of inflammation, complete blood counts (CBCs) were performed. CBC indicators were within the normal range for all tested CAF dogs; thus, no sign of serious inflammation or infection was detectable by CBC analysis (data not shown). Although hemoglobin levels were lower in CAF dogs belonging to the far cluster than in those in the near cluster, we did not identify any bacterial taxa associated with hemoglobin levels, nor with any of the analyzed blood inflammatory markers.

In sum, there is an inverse correlation between *H. canis* abundance and the number of fistulas. In addition to the bacterial taxa associated with disease status, CAF dogs exhibiting microarchitectural changes related to inflammation also exhibit decreased *R. torques* abundance. Furthermore, CAF dogs presenting microarchitectural defects combined with immune cell infiltration in the lamina propria have decreased abundance of *Subdoligranulum unclassified* and *Paraprevotella unclassified*.

### Microbial gene pathways change in accordance with disease status

**Figure 4.**
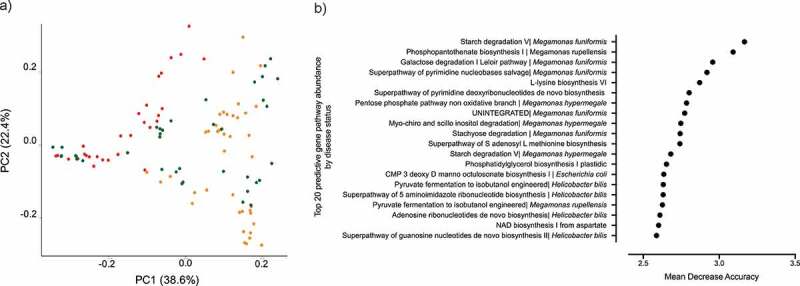
Microbial gene pathway analysis. A) Bray Curtis dissimilarity index beta diversity plot of dogs samples colored by clustering assignation: HC dogs (green) and CAF dogs belonging to the near (orange) or far (red) cluster. Bacterial pathways were quantified by HUMAnN2 and samples were plotted based on principal component analysis. B) The top 20 gene pathways that most strongly distinguish between CAF-dog samples and HC dog samples, identified using Random Forest.

The genomic content of the microbiota was determined using HUMAnN2.^54^ After filtering for gene pathways that were not present in at least 5% of the samples, we performed clustering analysis on 1,532 filtered pathways. Clustering of the gene pathway abundances separated CAF dogs belonging to either the near cluster or the far cluster from HC dogs (Figure 4A, PERMANOVA, R^2^ = 0.19, *p* value = .001).

Random Forest was used to identify microbial gene pathways that best distinguished HC dogs from CAF dogs, predicting differences with an OOB estimate of error rate of 6.48% (Figure 4B). As expected, most of the identified gene pathways originated from bacterial genera that were found to be predictive of disease status, specifically, *Megamonas, Bacteroides, and Helicobacter*.

Altogether, the metabolic capacity of the microbiome varied along with disease status or cluster assignation. Hence, most of the discriminant gene pathways belong exclusively to bacterial taxa depleted in CAF dogs. Although it is uncertain that this phenomenon causes or is a consequence of disease, characterization of these changes will enlighten our understanding of the microbial dynamics in disease.

## Discussion

Our understanding of the pathophysiology of pCD is not complete albeit their high prevalence and burden for patients with CD. The major challenge to study the pathogenesis of this manifestation is the absence of relevant animal models. Here, we characterized the microbiome of dogs suffering from CAF in order to explore the usefulness of dogs as models for pCD, a serious complication of CD that represents an unmet clinical need.

Other studies have aimed to characterize the microbiome composition of dogs with IBD with varying symptoms and from different breeds^55-58^ and their similarities with those from humans with IBD.^46^ However, this is the first study that aims to describe the similarities of dogs suffering from a specific clinical manifestation: canine furunculosis (CAF), to compare it to those from humans with CD. Moreover, we only included in the study German Shepherd Dogs, a breed susceptible to IBD with genetic polymorphisms comparable to those found in their IBD human counterpart.^59,60^

Interestingly, we found that similar to the microbiome of pediatric^44^ and adult^50^ patients with IBD, the microbiomes of CAF dogs can be either healthy-like or dysbiotic. The most recent Human Microbiome Project (HMP) study including 132 IBD patients and 2,965 stool samples collected over a year showed the same bi-modal microbiome pattern among patients with IBD.^50^ Specifically, the authors found that differences between dysbiotic and healthy-like microbiomes were more pronounced in patients with CD. Moreover, dysbiotic microbiomes in patients with CD were characterized by depletion of obligate anaerobes and enrichment of facultative-anaerobes.^50^ Similarly, we found that decreased abundance of obligate anaerobes such as *Megamonas* species and *P. copri* and increased abundance of facultative-anaerobes such as *E. coli* distinguish between samples from CAF dogs with a dysbiotic microbiome and those from HC dogs.

*Megamonas* is a common gut commensal microbe of carnivore animals. Increased abundance of *Megamonas* has been found in healthy cats^61^ and in dogs consuming inulin-rich diets.^62^ Few studies have shown the relevance of *Megamonas* in human health.^63,64^ *Megamonas* species are important propionate and acetate producers.^65,66^ Thus, based on these results, a greater emphasis on *Megamonas*, and its potential impact on gastrointestinal health, may be justified in the future.

Moreover, as seen in CAF dogs with a dysbiotic microbiome, pediatric patients with CD exhibiting a dysbiotic microbiome harbored significantly less abundance of *P. copri* compared to healthy control individuals.^44^ In the HMP study, however, abundance of *P. copri* was not significantly different between CD patients and healthy controls but the latter showed an expansion and relaxation of *P. copri* abundance over time. This organism is of particular interest given that it has been long associated with health but also more recently with new-onset of rheumatoid arthritis.^67^ Thus, further research on the role of *P. copri* in IBD is needed.

In the HMP study, the depleted anaerobe species in the far dysbiotic cluster were *Faecalibacterium praustnizii* and *Ruminococcus hominis*.^50^ Thus, although depleted bacterial species might not overlap between CAF and CD, we do observe a microbiome composition trend, namely a dysbiotic microbiome characterized by depletion of obligate anaerobes, that is similar between the two diseases.

On the other hand, we did find that increased abundance of *E.coli* overlaps between CAF and CD. Increased abundance of *Proteobacteria*, specifically *E. coli*, has been definitively demonstrated in patients with CD.^68–72^ In addition, the HMP study reported that CD patients exhibiting a dysbiotic microbiome harbor increased abundance of *E. coli*.^50^

We also found an enrichment of *Bacteroides vulgatus* in the far dysbiotic cluster. Increased abundance of *B. vulgatus* has previously been observed in dogs^46,56-58^ and in patients with IBD.^73,74^ *Bacteroides* species, namely *B. vulgatus*, are thought to be responsible for the development of inflammation and aberrant immune response in the gut.^75–79^ Therefore, these two strains seemed to be favored in the context of CAF and CD.

Concomitantly with what has been observed in patients with CD, we found that dysbiotic microbiomes are associated with host independent markers of disease.^50^ Here, CAF dogs with dysbiotic microbiomes tend to have a higher number of fistulas. We also found that markers of inflammation such as microarchitectural changes and immune cell infiltration in the lamina propria were negatively correlated with the abundance of *Ruminococcus torques, Subdoligranulum unclassified*, and *Paraprevotella unclassified*. Of note, *Ruminococcus torques* is a butyrate-producing *Clostridia* shown to induce anti-inflammatory activity and is known to be decreased in CD.^44,48,52,53^ Similarly, the HMP study^50^ established that *Subdoligranulum* species – also with butyrate-producing capacities – were both reduced in dysbiotic CD patients and associated dysregulation of IBD-linked metabolites (i.e., bile acids, carnitines, and polyunsaturated fatty acids).

In sum, we found that dogs suffering from CAF exhibit gut bacterial community structures that resemble those observed in CD patients. Moreover, we found overlapping bacteria species and their metabolic capacity associated with dysbiosis in both CAF and human CD. Therefore, we propose to use dogs suffering from CAF as a surrogate model to: (1) study pCD to further elucidate the role of the microbiome in this clinical complication, and (2) explore the pre-clinical efficacy of microbiome-centered therapeutics to treat pCD.

## Methods and materials

### Subjects and sample collection

All dogs in this study were GSD that had not received antibiotic treatment within the preceding 3 months and were treated with a special diet (novel protein or hypoallergenic) for at least 8 weeks without clinical response. CAF dogs were recruited from Cummings School of Veterinary Medicine at Tufts University. Inclusion criteria included German Shepard dogs with a clinical diagnosis of perianal fistulas with clinical signs of tenesmus, dyschezia, and partial or complete relapse from cyclosporine A therapy. HC dogs were selected based on the absence of perianal fistulas, nonexistence of diarrhea or adverse gastrointestinal symptoms, and lack of immunosuppressant treatment. Dog information including age, weight, gender, number of fistulas, treatment, and antibiotic usage was obtained from clinical records.

Naturally passed feces were collected daily for a period of 1 week from eight HC dogs and from 12 CAF dogs. Dog owners were provided with tubes (OMNI•gene-GUT| OMR-200, DNAgenotek, Canada) for sample collection and were instructed to place a clean pad on the surface before dogs’ bowel movement to avoid sample contamination from soil, grass, or other surfaces. Samples were freshly collected into the tubes provided. Samples were stored at room temperature until they were delivered at the next clinical appointment, which occurred within 2 weeks of sampling. During that next appointment, biopsies from the distal colon and blood samples were obtained from all CAF dogs for histopathological analysis and CBC, respectively. To determine colonic inflammation, an experienced pathologist blind-scored the biopsies based on the infiltration of eosinophils, lymphocytes, or plasma cells in the lamina propria and morphological changes, including crypt dilation/distortion, fibrosis/atrophy, or crypt hyperplasia, as previously described.^80^

### DNA isolation and sequencing

DNA isolation was performed using the MagAttract PowerSoil DNA Kit (Qiagen, # 27100-4-EP) on Eppendorf epMotion 5075 liquid handlers following the manufacturer’s instructions. DNA sequencing libraries were prepared using the Nextera XT DNA Library Preparation Kit (Illumina, #FC-131-1096) and were sequenced on the Illumina NextSeq 500 platform as 150-nt paired-end reads. We obtained an average of 3.8 M reads per sample (minimum: 1 M reads and maximum: 7.7 M reads). Read data were quality trimmed and filtered of host DNA using KneadData (https://bitbucket.org/biobakery/kneaddata/wiki/Home) against a prebuilt bowtie2 index for *Canis familiaris* reference genome, build 3.1.

### Metagenomic profiling

Community composition was profiled using MetaPhlan2^81^ to determine the per sample relative abundance of species composition of bacterial, fungal, parasitic, and viral genomes per sample. Then, to assess the content of metabolic and functional genes and metabolic pathways we used HUMAnN2.^54^

### Statistical analysis

For analysis, we considered only taxa that were detected in at least 10% of samples and had a relative abundance of at 0.2% or greater in at least one sample. Community patterns were analyzed using partitioning around medoids with the estimation of the number of clusters (PAMK) to find the optimal number of clusters as performed previously^44^ and visualized after multidimensional scaling (MDS). Clustering analyses and visualizations were performed in Phyloseq 1.26.1 and the R package cluster v1.4–1 to estimate microbiome patterns using PAMK with optimum average silhouette width.^82,83^ Briefly, the PAM algorithm is based on the search for “k” representative objects or k-medoids among the observations of the data set. In k-medoids clustering, each cluster is represented by one of the data point in the cluster. These points are named cluster medoids. After finding a set of k-medoids, clusters are constructed by assigning each observation to the nearest medoid. To estimate the optimal number of clusters, we estimated the average silhouette method. Here, we compute the PAM algorithm using different values of clusters k. Next, the average cluster silhouette is calculated according to the number of clusters. A high average silhouette width indicates a good clustering. The optimal number of clusters k is the one that maximizes the average silhouette over a range of possible values for k.

Shannon diversity index (alpha diversity) and Bray Curtis dissimilarity (beta diversity) were calculated using QIIME2 1.8.9. To assess the significance of differences in diversity metrics between samples according to either disease status or cluster assignation, we used Kruskal–Wallis, PERMANOVA, ADONIS, and Mantel tests. Determinant bacterial species were identified by the robust algorithms gneiss^47^ and ANCOM^84^ implemented in QIIME2.

In gneiss, we used linear-mixed models (LME) to account for the random effect of patients with multiple samples. LME are used to test the relationship between a single response variable and one or more independent variables, where observations are made across dependent samples. We then evaluated the correlations (with FRD correction) of 61 bacterial taxa with each variable separately, specifically disease status (CAF vs HC), cluster assignation (CAF near cluster, CAF far cluster, and HC), or clinical manifestations (number of fistulas, crypt hyperplasia, crypt dilation/distortion, fibrosis/atrophy). To understand the direction of dominance found in gneiss we performed Spearman correlations of the determinant bacterial taxa with the clinical manifestation of interest.

Additionally, we use ANCOM adjusting for dependent repeated measurements.^84^ For ANCOM, we analyzed 61 bacterial taxa, and only bacterial taxa reporting W > 45 were considered significant.

Finally, we used Random Forest (R package, randomForest 4.6–14),^6,8^a supervised learning algorithm, to identify gene pathways that discriminated samples by disease status as previously described.^44^

## Data Availability

Sequence files for all samples used in this study have been deposited in the NCBI SRA (SRA: SRP191145 and Bioproject PRJNA53120). Metadata and MetaPhlan tables – with corresponding taxonomic classifications – have been included as Additional files 1 and 2.

## Abbreviations

ANCOM: Analysis of Composition of Microbiomes
CD: Crohn’s disease
CAF: canine anal furunculosis
HC: Healthy controls
IBD: Inflammatory Bowel Diseases

## References

[1] Panes J, Reinisch W, Rupniewska E, Khan S, Forns J, Khalid JM, Bojic D, Patel H. Burden and outcomes for complex perianal fistulas in Crohn’s disease: systematic review. World Journal of Gastroenterology: WJG. 2018;24:4821–15. doi:10.3748/wjg.v24.i42.4821. 30479468 PMC6235801

[2] Kelley KA, Kaur T, Tsikitis VL. Perianal Crohn’s disease: challenges and solutions. Clin Exp Gastroenterol. 2017;10:39–46. doi:10.2147/CEG.S108513. 28223835 PMC5308478

[3] Beaugerie L, Seksik P, Nion-Larmurier I, Gendre JP, Cosnes J. Predictors of Crohn’s disease. Gastroenterology. 2006;130:650–656. doi:10.1053/j.gastro.2005.12.019. 16530505

[4] Cleynen I, González JR, Figueroa C, Franke A, McGovern D, Bortlík M, Crusius BJA, Vecchi M, Artieda M, Szczypiorska M, *et al*. Genetic factors conferring an increased susceptibility to develop Crohn’s disease also influence disease phenotype: results from the IBDchip European Project. Gut. 2013;62:1556–1565. doi:10.1136/gutjnl-2011-300777. 23263249

[5] Schnitzler F, Friedrich M, Wolf C, Stallhofer J, Angelberger M, Diegelmann J, Olszak T, Tillack C, Beigel F, Göke B, *et al*. The NOD2 Single nucleotide polymorphism rs72796353 (IVS4+10 A>C) is a predictor for perianal fistulas in patients with Crohn’s disease in the absence of other NOD2 mutations. PLoS One. 2015;10:e0116044. doi:10.1371/journal.pone.0116044. 26147989 PMC4493062

[6] Ruffolo C, Scarpa M, Faggian D, Romanato G, De Pellegrin A, Filosa T, Prando D, Polese L, Scopelliti M, Pilon F, *et al*. Cytokine network in chronic perianal Crohn’s disease and indeterminate colitis after colectomy. J Gastrointest Surg. 2007;11:16–21. doi:10.1007/s11605-006-0021-y. 17390181

[7] Mennigen R, Heptner B, Senninger N, Rijcken E. Temporary fecal diversion in the management of colorectal and perianal Crohn’s disease. Gastroenterol Res Pract. 2015;2015(286315). doi:10.1155/2015/286315. PMC4305613 25649893

[8] Rehg KL, Sanchez JE, Krieger BR, Marcet JE. Fecal diversion in perirectal fistulizing Crohn’s disease is an underutilized and potentially temporary means of successful treatment. Am Surg. 2009;75:715–718. 19725296

[9] Zhang FM, Wang HG, Wang M, *et al*. Fecal microbiota transplantation for severe enterocolonic fistulizing Crohn’s disease. World J Gastroenterol. 2013;19:7213–7216. doi:10.3748/wjg.v19.i41.7213. 24222969 PMC3819561

[10] Panes J, Rimola J. Perianal fistulizing Crohn’s disease: pathogenesis, diagnosis and therapy. Nat Rev Gastroenterol Hepatol. 2017. doi:10.1038/nrgastro.2017.104. 28790453

[11] Ferrer L, Kimbrel EA, Lam A, Falk EB, Zewe C, Juopperi T, Lanza R, Hoffman A. Treatment of perianal fistulas with human embryonic stem cell-derived mesenchymal stem cells: a canine model of human fistulizing Crohn’s disease. Regen Med. 2016;11:33–43. doi:10.2217/rme.15.69. 26387424

[12] Hoffman AM, Dow SW. Concise review: stem cell trials using companion animal disease models. Stem Cells. 2016;34:1709–1729. doi:10.1002/stem.2377. 27066769

[13] Sandborn WJ, Fazio VW, Feagan BG, Hanauer SB. American gastroenterological association clinical practice, C. AGA technical review on perianal Crohn’s disease. Gastroenterology. 2003;125:1508–1530. doi:10.1016/j.gastro.2003.08.025. 14598268

[14] Taxonera C, Schwartz DA, Garcia-Olmo D. Emerging treatments for complex perianal fistula in Crohn’s disease. World Journal of Gastroenterology: WJG. 2009;15:4263–4272. doi:10.3748/wjg.15.4263. 19750568 PMC2744181

[15] Jamieson PM, Simpson JW, Kirby BM, Else RW. Association between anal furunculosis and colitis in the dog: preliminary observations. J Small Anim Pract. 2002;43:109–114. doi:10.1111/j.1748-5827.2002.tb00039.x. 11916054

[16] Cain CL. Canine perianal fistulas: clinical presentation, pathogenesis, and management. Vet Clin North Am Small Anim Pract. 2019;49:53–65. doi:10.1016/j.cvsm.2018.08.006. 30213533

[17] Hardie RJ, Gregory SP, Tomlin J, Sturgeon C, Lipscomb V, Ladlow J. Cyclosporine treatment of anal furunculosis in 26 dogs. J Small Anim Pract. 2005;46:3–9. 10.1111/j.1748-5827.2005.tb00267.x. 15682733 10.1111/j.1748-5827.2005.tb00267.x

[18] Gold SL, Cohen-Mekelburg S, Schneider Y, Steinlauf A. Perianal fistulas in patients with Crohn’s disease, part 1: current medical management. Gastroenterol Hepatol (N Y). 2018;14:470–481. 30302062 PMC6170888

[19] Adam P, Patterson KLC. Managing anal furunculosis in dogs. Internal Medicine Compendium. 2005;27. https://pdfs.semanticscholar.org/1fe4/5115545e42e8133503e87a1a4343c46ce4ff.pdf.

[20] Tisdall PL, Hunt GB, Beck JA, Malik R. Management of perianal fistulae in five dogs using azathioprine and metronidazole prior to surgery. Aust Vet J. 1999;77:374–378. doi:10.1111/j.1751-0813.1999.tb10307.x. 10812402

[21] Stanley BJ, Hauptman JG. Long-term prospective evaluation of topically applied 0.1% tacrolimus ointment for treatment of perianal sinuses in dogs. J Am Vet Med Assoc. 2009;235:397–404. doi:10.2460/javma.235.4.397. 19681720

[22] Levine A, Rhodes JM, Lindsay JO, Abreu MT, Kamm MA, Gibson PR, Gasche C, Silverberg MS, Mahadevan U, Boneh RS, *et al*. Dietary Guidance for Patients with Inflammatory Bowel Disease from the International Organization for the study of inflammatory bowel disease. Clin Gastroenterol Hepatol. 2020. doi:10.1016/j.cgh.2020.01.046. 32068150

[23] Marlow G, Ellett S, Ferguson IR, Zhu S, Karunasinghe N, Jesuthasan AC, Han D, Fraser AG, Ferguson LR. Transcriptomics to study the effect of a Mediterranean-inspired diet on inflammation in Crohn’s disease patients. Hum Genomics. 2013;7(24). doi:10.1186/1479-7364-7-24. PMC4174666 24283712

[24] Suskind DL, Wahbeh G, Gregory N, Vendettuoli H, Christie D. Nutritional therapy in pediatric Crohn disease: the specific carbohydrate diet. J Pediatr Gastroenterol Nutr. 2014;58:87–91. doi:10.1097/MPG.0000000000000103. 24048168

[25] Olendzki BC, Silverstein TD, Persuitte GM, Ma Y, Baldwin KR, Cave D. An anti-inflammatory diet as treatment for inflammatory bowel disease: a case series report. Nutr J. 2014;13(5). doi:10.1186/1475-2891-13-5. PMC3896778 24428901

[26] Proverbio D, Perego R, Spada E, Ferro E. Prevalence of adverse food reactions in 130 dogs in Italy with dermatological signs: a retrospective study. J Small Anim Pract. 2010;51:370–374. doi:10.1111/j.1748-5827.2010.00951.x. 20536692

[27] Lombardi RL, Marino DJ. Long-term evaluation of canine perianal fistula disease treated with exclusive fish and potato diet and surgical excision. J Am Anim Hosp Assoc. 2008;44:302–307. doi:10.5326/0440302. 18981195

[28] Harkin KR, Walshaw R, Mullaney TP. Association of perianal fistula and colitis in the German shepherd dog: response to high-dose prednisone and dietary therapy. J Am Anim Hosp Assoc. 1996;32:515–520. doi:10.5326/15473317-32-6-515. 8906729

[29] Day MJ, W. BMQ. Pathology of surgically resected tissue from 305 cases of anal furunculosis in the dog. Journal of Small Animal Practice. 1992;33:583–589. 10.1111/j.1748-5827.1992.tb01062.x.

[30] Killingsworth CR, Walshaw R, Dunstan RW, Rosser EJ Jr. Bacterial population and histologic changes in dogs with perianal fistula. Am J Vet Res. 1988;49:1736–1741. 3189990

[31] Kathrani A, House A, Catchpole B, Murphy A, German A, Werling D, Allenspach K. Polymorphisms in the TLR4 and TLR5 gene are significantly associated with inflammatory bowel disease in German shepherd dogs. PLoS One. 2010;5(e15740). doi:10.1371/journal.pone.0015740. PMC3009732 21203467

[32] Kathrani A, Lee H, White C, Catchpole B, Murphy A, German A, Werling D, Allenspach K. Association between nucleotide oligomerisation domain two (Nod2) gene polymorphisms and canine inflammatory bowel disease. Vet Immunol Immunopathol. 2014;161:32–41. doi:10.1016/j.vetimm.2014.06.003. 25017709

[33] Bruchim Y, Elad D, Klainbart S. Disseminated aspergillosis in two dogs in Israel. Mycoses. 2006;49:130–133. doi:10.1111/j.1439-0507.2006.01168.x. 16466447

[34] Day MJ, Penhale WJ, Kabay MJ, Robinson WF, Huxtable CRR, Eger CE, Shaw SE, Mills JN, Wyburn RS. Disseminated aspergillosis in dogs. Aust Vet J. 1986;63:55–59. 10.1111/j.1751-0813.1986.tb02924.x. 3964146 10.1111/j.1751-0813.1986.tb02924.x

[35] Cook E, Meler E, Garrett K, Long H, Mak K, Stephens C, Thompson A. Disseminated Chrysosporium infection in a German shepherd dog. Med Mycol Case Rep. 2015;10:29–33. doi:10.1016/j.mmcr.2016.01.002. 26937338 PMC4769606

[36] Pastor J, Pumarola M, Cuenca R, Lavin S. Systemic aspergillosis in a dog. Vet Rec. 1993;132:412–413. 10.1136/vr.132.16.412. 8488661 10.1136/vr.132.16.412

[37] Rosser EJ Jr. German shepherd dog pyoderma: a prospective study of 12 dogs. J Am Anim Hosp Assoc. 1997;33:355–363. doi:10.5326/15473317-33-4-355. 9204474

[38] Miller WH. Deep pyoderma in two German shepherd dogs associated with a cell mediated immunodeficiency. J Am Anim Hosp Assoc. 1991;27.

[39] Vail DM, MacEwen EG. Spontaneously occurring tumors of companion animals as models for human cancer. Cancer Invest. 2000;18:781–792. 10.3109/07357900009012210. 11107448 10.3109/07357900009012210

[40] Cadieu E, Ostrander EA. Canine genetics offers new mechanisms for the study of human cancer. Cancer Epidemiol Biomarkers Prev. 2007;16:2181–2183. doi:10.1158/1055-9965.EPI-07-2667. 17982116

[41] Jonasdottir TJ, Mellersh CS, Moe L, Heggebo R, Gamlem H, Ostrander EA, Lingaas F. Genetic mapping of a naturally occurring hereditary renal cancer syndrome in dogs. Proc Natl Acad Sci U S A. 2000;97:4132–4137. doi:10.1073/pnas.070053397. 10759551 PMC18172

[42] Overall KL. Natural animal models of human psychiatric conditions: assessment of mechanism and validity. Prog Neuropsychopharmacol Biol Psychiatry. 2000;24:727–776. 10.1016/S0278-5846(00)00104-4. 11191711 10.1016/s0278-5846(00)00104-4

[43] Coelho LP, Kultima JR, Costea PI, Fournier C, Pan Y, Czarnecki-Maulden G, Hayward MR, Forslund SK, Schmidt TSB, Descombes P, *et al*. Similarity of the dog and human gut microbiomes in gene content and response to diet. Microbiome. 2018;6(72). doi:10.1186/s40168-018-0450-3 PMC5907387 29669589

[44] Lewis JD, Chen E, Baldassano R, Otley A, Griffiths A, Lee D, Bittinger K, Bailey A, Friedman E, Hoffmann C, *et al*. Inflammation, antibiotics, and diet as environmental stressors of the gut microbiome in pediatric Crohn’s disease. Cell Host Microbe. 2015;18:489–500. doi:10.1016/j.chom.2015.09.008. 26468751 PMC4633303

[45] Halfvarson J, Brislawn CJ, Lamendella R, Vázquez-Baeza Y, Walters WA, Bramer LM, D’Amato M, Bonfiglio F, McDonald D, Gonzalez A, *et al*. Dynamics of the human gut microbiome in inflammatory bowel disease. Nat Microbiol. 2017;2(17004). doi:10.1038/nmicrobiol.2017.4 PMC5319707 28191884

[46] Vazquez-Baeza Y, Hyde ER, Suchodolski JS, Knight R. Dog and human inflammatory bowel disease rely on overlapping yet distinct dysbiosis networks. Nat Microbiol. 2016;1(16177). doi:10.1038/nmicrobiol.2016.177. 27694806

[47] Morton JT, Sanders J, Quinn RA, McDonald D, Gonzalez A, Vázquez-Baeza Y, Navas-Molina JA, Song SJ, Metcalf JL, Hyde ER, *et al*. Balance trees reveal microbial niche differentiation. mSystems. 2017;2. doi:10.1128/mSystems.00162-16. PMC5264246 28144630

[48] Gevers D, Kugathasan S, Denson L, Vázquez-Baeza Y, Van Treuren W, Ren B, Schwager E, Knights D, Song S, Yassour M, *et al*. The treatment-naive microbiome in new-onset Crohn’s disease. Cell Host Microbe. 2014;15:382–392. doi:10.1016/j.chom.2014.02.005. 24629344 PMC4059512

[49] Frank DN, St. Amand AL, Feldman RA, Boedeker EC, Harpaz N, Pace NR. Molecular-phylogenetic characterization of microbial community imbalances in human inflammatory bowel diseases. Proc Natl Acad Sci U S A. 2007;104:13780–13785. doi:10.1073/pnas.0706625104. 17699621 PMC1959459

[50] Lloyd-Price J, Arze C, Ananthakrishnan AN, Schirmer M, Avila-Pacheco J, Poon TW, Andrews E, Ajami NJ, Bonham KS, Brislawn CJ, *et al*. Multi-omics of the gut microbial ecosystem in inflammatory bowel diseases. Nature. 2019;569:655–662. doi:10.1038/s41586-019-1237-9. 31142855 PMC6650278

[51] Robert J, Washabau MJD. Canine and feline gastroenterology. USA: Elsevier Saunders; 2013.

[52] Takahashi K, Nishida A, Fujimoto T, Fujii M, Shioya M, Imaeda H, Inatomi O, Bamba S, Andoh A, Sugimoto M, *et al*. Reduced abundance of butyrate-producing bacteria species in the fecal microbial community in Crohn’s disease. Digestion. 2016;93:59–65. doi:10.1159/000441768. 26789999

[53] Joossens M, Huys G, Cnockaert M, De Preter V, Verbeke K, Rutgeerts P, Vandamme P, Vermeire S. Dysbiosis of the faecal microbiota in patients with Crohn’s disease and their unaffected relatives. Gut. 2011;60:631–637. doi:10.1136/gut.2010.223263. 21209126

[54] Abubucker S, Segata N, Goll J, Schubert AM, Izard J, Cantarel BL, Rodriguez-Mueller B, Zucker J, Thiagarajan M, Henrissat B, *et al*. Metabolic reconstruction for metagenomic data and its application to the human microbiome. PLoS Comput Biol. 2012;8(e1002358). doi:10.1371/journal.pcbi.1002358 PMC3374609 22719234

[55] Guard BC, Barr JW, Reddivari L, Klemashevich C, Jayaraman A, Steiner JM, Vanamala J, Suchodolski JS. Characterization of microbial dysbiosis and metabolomic changes in dogs with acute diarrhea. PLoS One. 2015;10(e0127259). doi:10.1371/journal.pone.0127259. PMC4441376 26000959

[56] Suchodolski JS, Xenoulis PG, Paddock CG, Steiner JM, Jergens AE. Molecular analysis of the bacterial microbiota in duodenal biopsies from dogs with idiopathic inflammatory bowel disease. Vet Microbiol. 2010;142:394–400. doi:10.1016/j.vetmic.2009.11.002. 19959301

[57] Suchodolski JS, Dowd SE, Wilke V, Steiner JM, Jergens AE. 16S rRNA gene pyrosequencing reveals bacterial dysbiosis in the duodenum of dogs with idiopathic inflammatory bowel disease. PLoS One. 2012;7(e39333). doi:10.1371/journal.pone.0039333. PMC3376104 22720094

[58] Minamoto Y, Otoni CC, Steelman SM, Büyükleblebici O, Steiner JM, Jergens AE, Suchodolski JS. Alteration of the fecal microbiota and serum metabolite profiles in dogs with idiopathic inflammatory bowel disease. Gut Microbes. 2015;6(33–47). doi:10.1080/19490976.2014.997612. PMC4615558 25531678

[59] Massey J, Short AD, Catchpole B, House A, Day MJ, Lohi H, Ollier WER, Kennedy LJ. Genetics of canine anal furunculosis in the German shepherd dog. Immunogenetics. 2014;66:311–324. doi:10.1007/s00251-014-0766-5. 24626934

[60] Peiravan A, Bertolini F, Rothschild MF, Simpson KW, Jergens AE, Allenspach K, Werling D. Genome-wide association studies of inflammatory bowel disease in German shepherd dogs. PLoS One. 2018;13(e0200685). doi:10.1371/journal.pone.0200685. PMC6054420 30028859

[61] Suchodolski JS, Foster ML, Sohail MU, Leutenegger C, Queen EV, Steiner JM, Marks SL. The fecal microbiome in cats with diarrhea. PLoS One. 2015;10(e0127378). doi:10.1371/journal.pone.0127378. PMC4437779 25992741

[62] Beloshapka AN, Dowd SE, Suchodolski JS, Steiner JM, Duclos L, Swanson KS. Fecal microbial communities of healthy adult dogs fed raw meat-based diets with or without inulin or yeast cell wall extracts as assessed by 454 pyrosequencing. FEMS Microbiol Ecol. 2013;84:532–541. doi:10.1111/1574-6941.12081. 23360519

[63] Zhang X, Shen D, Fang Z, Jie Z, Qiu X, Zhang C, *et al*. Human gut microbiota changes reveal the progression of glucose intolerance. PLoS One. 2013;8(e71108). doi:10.1371/journal.pone.0071108 PMC3754967 24013136

[64] Chen L, Wang W, Zhou R, Ng SC, Li J, Huang M, Zhou F, Wang X, Shen BA, Kamm M, *et al*. Characteristics of fecal and mucosa-associated microbiota in Chinese patients with inflammatory bowel disease. Medicine (Baltimore). 2014;93(e51). doi:10.1097/MD.0000000000000051 PMC4602441 25121355

[65] Zhao C, Dong H, Zhang Y, Li Y. Discovery of potential genes contributing to the biosynthesis of short-chain fatty acids and lactate in gut microbiota from systematic investigation in E. coli. NPJ Biofilms Microbiomes. 2019;5(19). doi:10.1038/s41522-019-0092-7. PMC6626047 31312512

[66] Polansky O, Sekelova Z, Faldynova M, Sebkova A, Sisak F, Rychlik I. Important metabolic pathways and biological processes expressed by chicken cecal microbiota. Appl Environ Microbiol. 2015;82:1569–1576. doi:10.1128/AEM.03473-15. 26712550 PMC4771310

[67] Scher JU, Sczesnak A, Longman RS, Segata N, Ubeda C, Bielski C, Rostron T, Cerundolo V, Pamer EG, Abramson SB, *et al*. Expansion of intestinal Prevotella copri correlates with enhanced susceptibility to arthritis. Elife. 2013;2(e01202). doi:10.7554/eLife.01202 PMC3816614 24192039

[68] Martinez C, Antolin M, Santos J, Torrejon A, Casellas F, Borruel N, Guarner F, Malagelada J-R. Unstable composition of the fecal microbiota in ulcerative colitis during clinical remission. Am J Gastroenterol. 2008;103:643–648. doi:10.1111/j.1572-0241.2007.01592.x. 18341488

[69] Baumgart M, Dogan B, Rishniw M, Weitzman G, Bosworth B, Yantiss R, Orsi RH, Wiedmann M, McDonough P, Kim SG, *et al*. Culture independent analysis of ileal mucosa reveals a selective increase in invasive Escherichia coli of novel phylogeny relative to depletion of Clostridiales in Crohn’s disease involving the ileum. Isme J. 2007;1:403–418. doi:10.1038/ismej.2007.52. 18043660

[70] Kotlowski R, Bernstein CN, Sepehri S, Krause DO. High prevalence of Escherichia coli belonging to the B2+D phylogenetic group in inflammatory bowel disease. Gut. 2007;56:669–675. doi:10.1136/gut.2006.099796. 17028128 PMC1942160

[71] Martin HM, Campbell BJ, Hart CA, Mpofu C, Nayar M, Singh R, Englyst H, Williams HF, Rhodes JM. Enhanced Escherichia coli adherence and invasion in Crohn’s disease and colon cancer. Gastroenterology. 2004;127:80–93. 10.1053/j.gastro.2004.03.054. 15236175 10.1053/j.gastro.2004.03.054

[72] Nishino K, Nishida A, Inoue R, Kawada Y, Ohno M, Sakai S, Inatomi O, Bamba S, Sugimoto M, Kawahara M, *et al*. Analysis of endoscopic brush samples identified mucosa-associated dysbiosis in inflammatory bowel disease. J Gastroenterol. 2018;53:95–106. doi:10.1007/s00535-017-1384-4. 28852861

[73] Fujita H, Eishi Y, Ishige I, Saitoh K, Takizawa T, Arima T, Koike M. Quantitative analysis of bacterial DNA from Mycobacteria spp., Bacteroides vulgatus, and Escherichia coli in tissue samples from patients with inflammatory bowel diseases. J Gastroenterol. 2002;37:509–516. doi:10.1007/s005350200079. 12162408

[74] Lucke K, Miehlke S, Jacobs E, Schuppler M. Prevalence of Bacteroides and Prevotella spp. in ulcerative colitis. J Med Microbiol. 2006;55:617–624. doi:10.1099/jmm.0.46198-0. 16585651

[75] Bloom SM, Bijanki V, Nava G, Sun L, Malvin N, Donermeyer D, Dunne W, Allen P, Stappenbeck T. Commensal Bacteroides species induce colitis in host-genotype-specific fashion in a mouse model of inflammatory bowel disease. Cell Host Microbe. 2011;9:390–403. doi:10.1016/j.chom.2011.04.009. 21575910 PMC3241010

[76] Bamba T, Matsuda H, Endo M, Fujiyama Y. The pathogenic role of Bacteroides vulgatus in patients with ulcerative colitis. J Gastroenterol. 1995;30:45–47. 8563888

[77] Breeling JL, Onderdonk AB, Cisneros RL, Kasper DL. Bacteroides vulgatus outer membrane antigens associated with carrageenan-induced colitis in guinea pigs. Infect Immun. 1988;56:1754–1759. 10.1128/IAI.56.7.1754-1759.1988. 3384476 10.1128/iai.56.7.1754-1759.1988 PMC259473

[78] Matsuda H, Fujiyama Y, Andoh A, Ushijima T, Kajinami T, Bamba T. Characterization of antibody responses against rectal mucosa-associated bacterial flora in patients with ulcerative colitis. J Gastroenterol Hepatol. 2000;15:61–68. doi:10.1046/j.1440-1746.2000.02045.x. 10719749

[79] Shiba T, Aiba Y, Ishikawa H, Ushiyama A, Takagi A, Mine T, Koga Y. The suppressive effect of bifidobacteria on bacteroides vulgatus, a putative pathogenic microbe in inflammatory bowel disease. Microbiol Immunol. 2003;47:371–378. doi:10.1111/j.1348-0421.2003.tb03368.x. 12906096

[80] Jergens AE, Evans RB, Ackermann M, Hostetter J, Willard M, Mansell J, Bilzer T, Wilcock B, Washabau R, Hall EJ, *et al*. Design of a simplified histopathologic model for gastrointestinal inflammation in dogs. Vet Pathol. 2014;51:946–950. doi:10.1177/0300985813511123. 24280943

[81] Truong DT, Franzosa EA, Tickle TL, Scholz M, Weingart G, Pasolli E, Tett A, Huttenhower C, Segata N. MetaPhlAn2 for enhanced metagenomic taxonomic profiling. Nat Methods. 2015;12:902–903. doi:10.1038/nmeth.3589. 26418763

[82] Rousseeuw P. Silhouettes: A graphical aid to the interpretation and validation of cluster analysis. J Comput Appl Math. 1987;20:53–65. 10.1016/0377-0427(87)90125-7.

[83] Kaufman L, A. R. PJ. Finding groups in data: an introduction to cluster analysis. USA and Canada: John Wiley & Sons, Inc; 1990.

[84] Mandal S, Van Treuren W, White RA, Eggesbo M, Knight R, Peddada SD. Analysis of composition of microbiomes: a novel method for studying microbial composition. Microb Ecol Health Dis. 2015;26(27663). doi:10.3402/mehd.v26.27663 PMC4450248 26028277

